# Frailty: the ideal target for prevention?

**DOI:** 10.1007/s41999-024-01014-w

**Published:** 2024-07-26

**Authors:** Michael Denkinger, Ivan Aprahamian

**Affiliations:** 1 Institute for Geriatric Research, Ulm University Medical Center at AGAPLESION Bethesda Ulm, 89073 Ulm, Germany; 2 Group of Investigation on Multimorbidity and Mental Health in Aging (GIMMA), Division of Geriatrics, Department of Internal Medicine, Faculty of Medicine of Jundiaí, São Paulo, Brazil

Frailty is highly prevalent. Its definition, however, has produced a lot of discussion, uncertainty, and disagreement over the years. Despite this, there are two things about frailty that are accepted by the whole research community: (1) as a state of increased vulnerability to any kind of physiological and psychological stressors, frailty is associated with numerous negative outcomes, such as functional decline, disability and ultimately death [1]; and (2) reversing frailty is possible but difficult [2, 3]. Reversing or preventing frailty clearly must, therefore, be of high interest to the community and public health stakeholders because of the associated outcomes. But, from a public health point of view, is it more important to show that frailty can be prevented or is it rather institutionalization or hospitalization? And, what is more relevant, primary prevention in non-frail older adults with the aim of not to become frail or secondary or even tertiary prevention in frail and disabled older adults not become institutionalized?

In this issue of European Geriatric Medicine, Eidam et al. analyzed randomized controlled trials of interventions aimed at preventing frailty in non-frail older adults (defined as robust or pre-frail individuals) [4]. Their findings suggest that physical exercise-based, and less so nutritional, interventions can lower the risk of developing frailty [4]. Prior research has primarily focused on the impact of interventions on frailty in frail or mixed non-frail/frail populations [2, 3, 5]. Eidam et al. excluded interventions that were primarily focusing on medication optimization but included those that used multifactorial interventions. They also exclude trials that did not have frailty measurements at follow-up or with primary outcomes such as grip strength as a proxy. Meta-analysis was only done on those studies that reported a dichotomous frailty endpoint as assessed by the concept of the physical frailty phenotype.

This strict methodological approach has the advantage of selecting those interventions that prove to be successful of preventing the development of physical and functional limitations. It is, therefore, no surprise that mostly exercise interventions demonstrate effectivity, which confirms other systematic reviews in mixed populations [2, 6]. Exercise interventions have also proven successful in a new systematic review by Crocker et al. that focuses on broader and more directly public health relevant outcomes such as living at home independently and activities of daily living [7]. They included studies with all kinds of complex interventions within the community that were implemented to sustain independence in older people. Results show that complex interventions work for (frail) older people only if they are truly multifactorial and, above all, if nutrition, physical training, and a medication review are combined.

Therefore, although for advancing Geriatric medicine, strict methodological approaches such as the one taken by Eidam et al. are important, results must be set into context. While the phenotypic frailty assessment mainly assesses functional status and highly correlates with sarcopenia, other scales have a much more multidimensional approach that also consider psychological, cognitive, or even social factors. This can hardly be summed to one single geriatric frailty syndrome [1, 8]. Moreover, indices like the cumulative and deficit-oriented frailty index (FI) even mostly reflect multi-morbidity and often inherits little functional aspects [9, 10].

To clearly demonstrate the value of geriatric medicine, we should, therefore, focus on endpoints that are well framed and that matter most to older people, to public health stakeholders and to politicians. Independent living could be one of the most relevant and, in opposite to institutionalization or hospitalization, it is not deficit-oriented. Although that might seem a petty, the Salutogenesis model, that was developed in the 80 s has shown, that it is not [11]. Positive language and capacity-oriented thinking will, alongside with disease-/deficit-prevention, set our mind frames toward strengthening resilience, robustness and intrinsic capacity (IC) [12].

In 2015, the World Health Organization (WHO) adopted IC, defined as ‘the composite of all the physical and mental capacities an individual can draw on as a new framework of healthy ageing [13]. WHO recommends improving IC to promote health aging. It is clear that all interventions that address IC also should reduce frailty and improve downstream outcomes such as higher independence or less disability. Trials, which focus on improving IC, therefore, have comparable elements to trials with other resource or deficit-oriented endpoints. In a systematic review with meta-analysis of multi-domain interventions for IC, the authors found that several interventions could improve or delay the decline of at least one component of IC [14]. In this review, successful studies, again, used resistive exercises and nutritional interventions with protein supplementation or balanced meals offered to participants. All studies that improved vitality implemented protein supplements and studies that improved mental health mostly had physical exercise and cognitive stimulating elements [14].

Future studies that are intended to improve IC could also build on the WHO-promoted integrated care for older adults (ICOPE) guidelines. A recent one, + AGIL, showed that a multi-domain & multiprofessional person-centered intervention according to ICOPE could improve physical performance while maintaining stable frailty characteristics in community-dwelling older adults. The + AGIL intervention protocol consisted of a frequent, but low level, mostly self-promoted, physical exercise program based on the VIVIFRAIL initiative, a healthy lifestyle promotion (especially concerning nutrition and sleep hygiene) using motivational interview, a medication review, and a specialty referral if cognitive impairment, depression or loneliness were detected. In a recent publication involving 194 participants from the program, improvement in physical performance (using the Short Physical Performance Battery test) was observed after 3 months (including a positive, non-significant trend after 6 months) [15] .

Yet still, the frailty syndrome should not at all be dismissed. Measuring frailty, especially within a screening process is already being used to identify older adults at need for a further work-up and to decide, whether and how far to go with certain, more harmful interventions [16]. Ideally, frailty screening with sensitive (and reasonably specific) instruments could help identify older adults at risk for adverse health outcomes in the community, before a certain harmful intervention or treatment and make them undergo a work-up with comprehensive geriatric assessment (CGA). CGA is evidence-based in several community and hospital-based settings and allows for derivation of appropriate interventions and monitoring outcome trajectories [17].

**Fig. 1:**
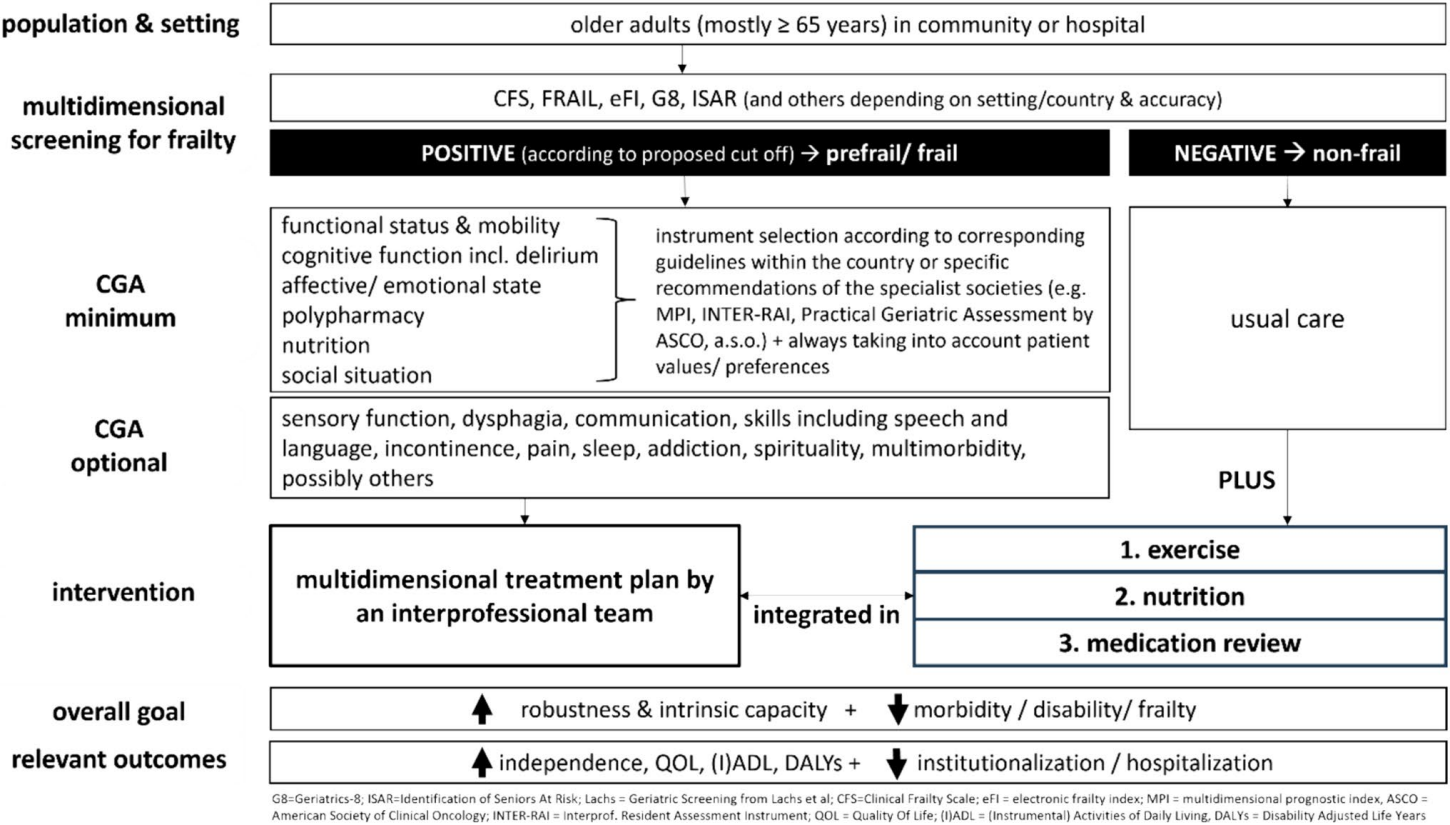
How frailty screening, CGA and primary prevention could go together. Primary prevention with exercise, nutrition support & medication (review) adapted to frailty levels for everybody and personalised CGA-based Geriatric treatment for prefrail and frail older people who need a differentiated approach

An important current step is certainly the forthcoming of the two first evidence & consensus-based guidelines for Comprehensive Geriatric Assessment in Germany (only hospitalized patients) and Italy (community & hospital) [18, 19] . In view of the rapidly increasing number of very old people and the associated rise in the prevalence of multi-morbidity and frailty worldwide, it is very important that strategies for maintaining autonomy and living-in-place are validated and implemented. A possible strategy has been detailed in Fig. 1, which combines findings from the above-mentioned studies as well as two new CGA guidelines, to propose a concept for a practical and beneficial algorithm.

While CGA would then be reserved for those older adults identified as pre-frail or frail and lead to multidimensional interventions directed toward certain deficits and resources (hearing or vision impairment, treatment of depression and pain, interventions on social networking and loneliness and others), exercise (with/without nutrition) and possible pharmacological interventions (treatment with primary prevention agents as well as medication review) should be available for both, non-frail and (pre)frail older people.

The algorithm in Fig. 1 could serve both, to prevent and reverse frailty as a mediating factor to achieve outcomes that matter most. While evidence is rising, it is now time to move on to implementation of complex interventions in our different health care systems—let’s get going!
